# Supplementary figures and images for: Integrated Metabolomic and Transcriptomic Analysis Reveals the Flavonoid Regulatory Network by Eutrema EsMYB90
Source: Int J Mol Sci. 2021 Aug 15;22(16):8751. doi: 10.3390/ijms22168751 (PMC8395869; doi:10.3390/ijms22168751)

**A**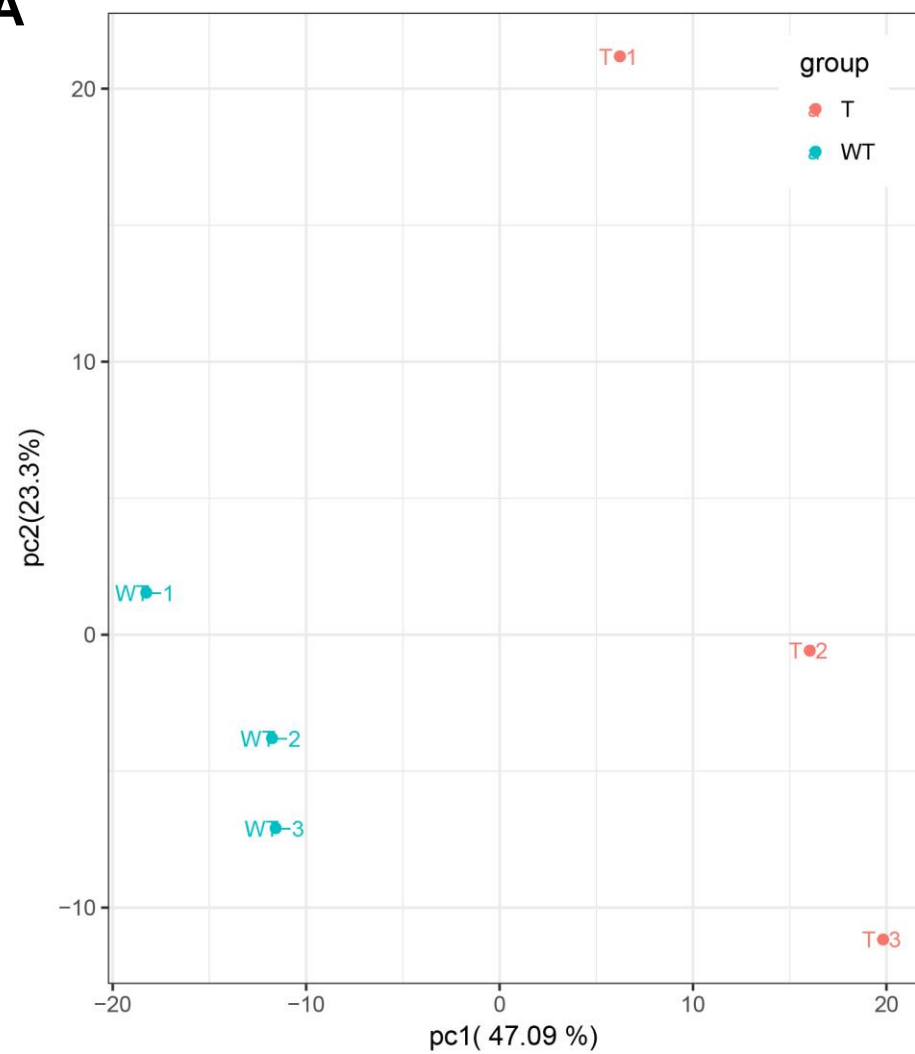**B**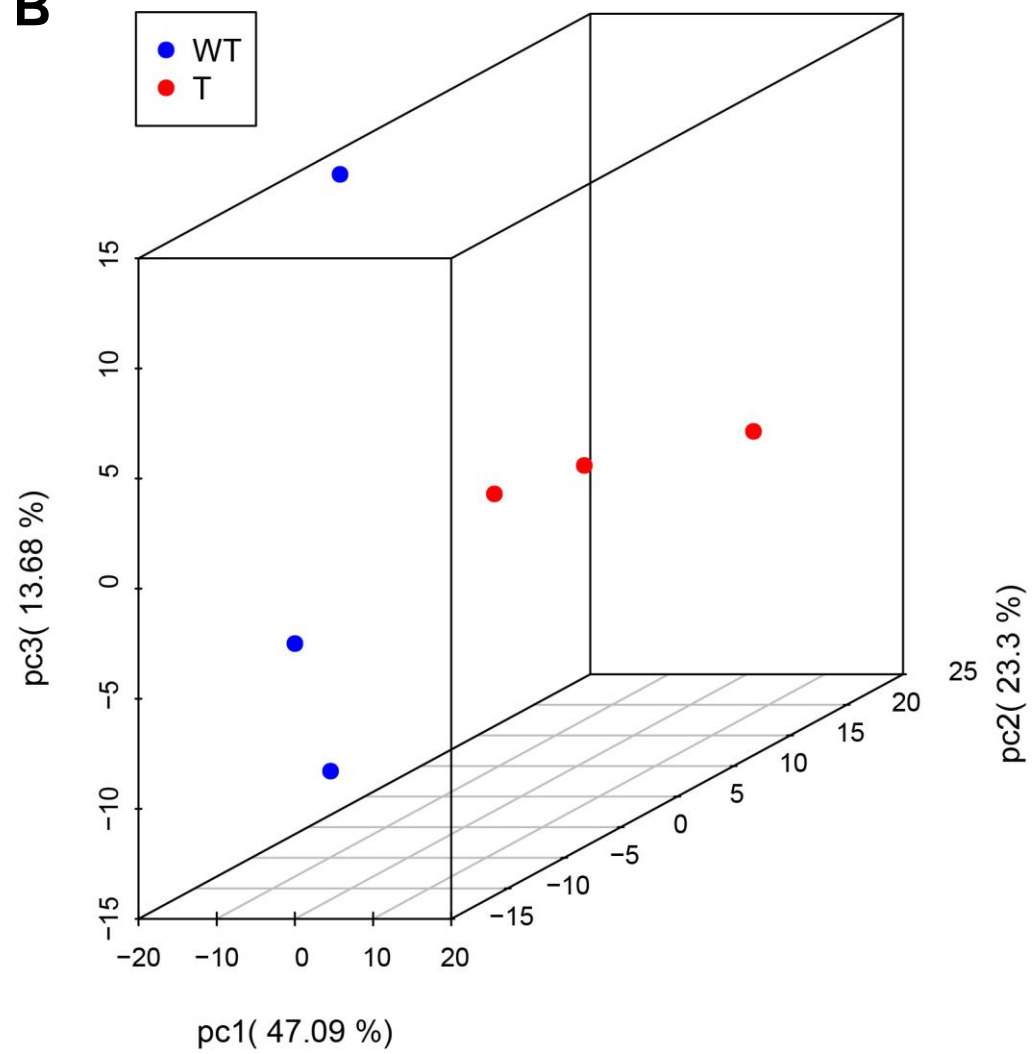

Supplement: Supplementary file 1 [file ijms-22-08751-s001.zip › 5 IJMS-Supplementary Files-8.3/Supplementary Figure S1 Principal component analysis in leaves of EsMYB90 transgenic tobacco and wild type.pdf]

**A**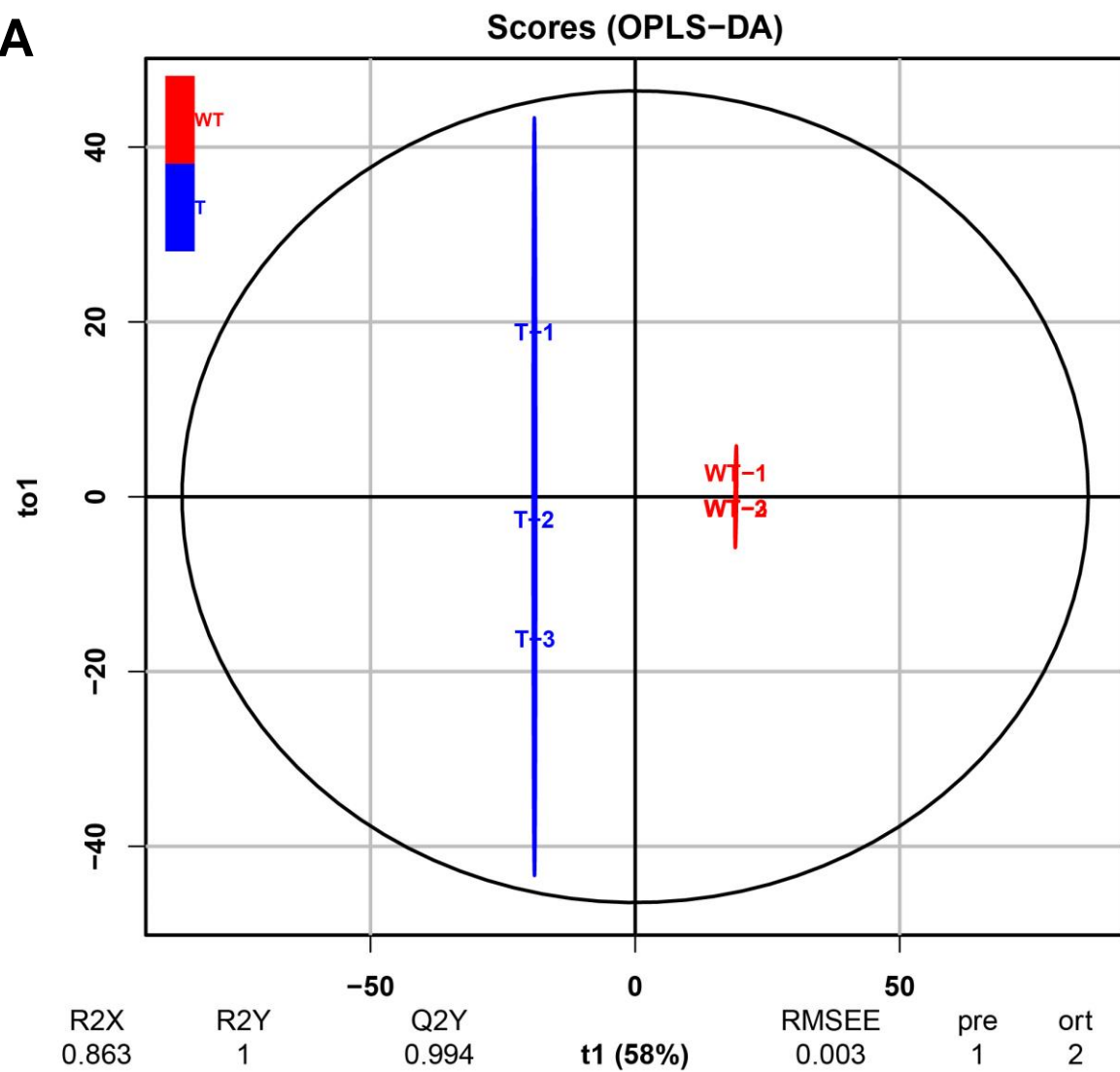**B**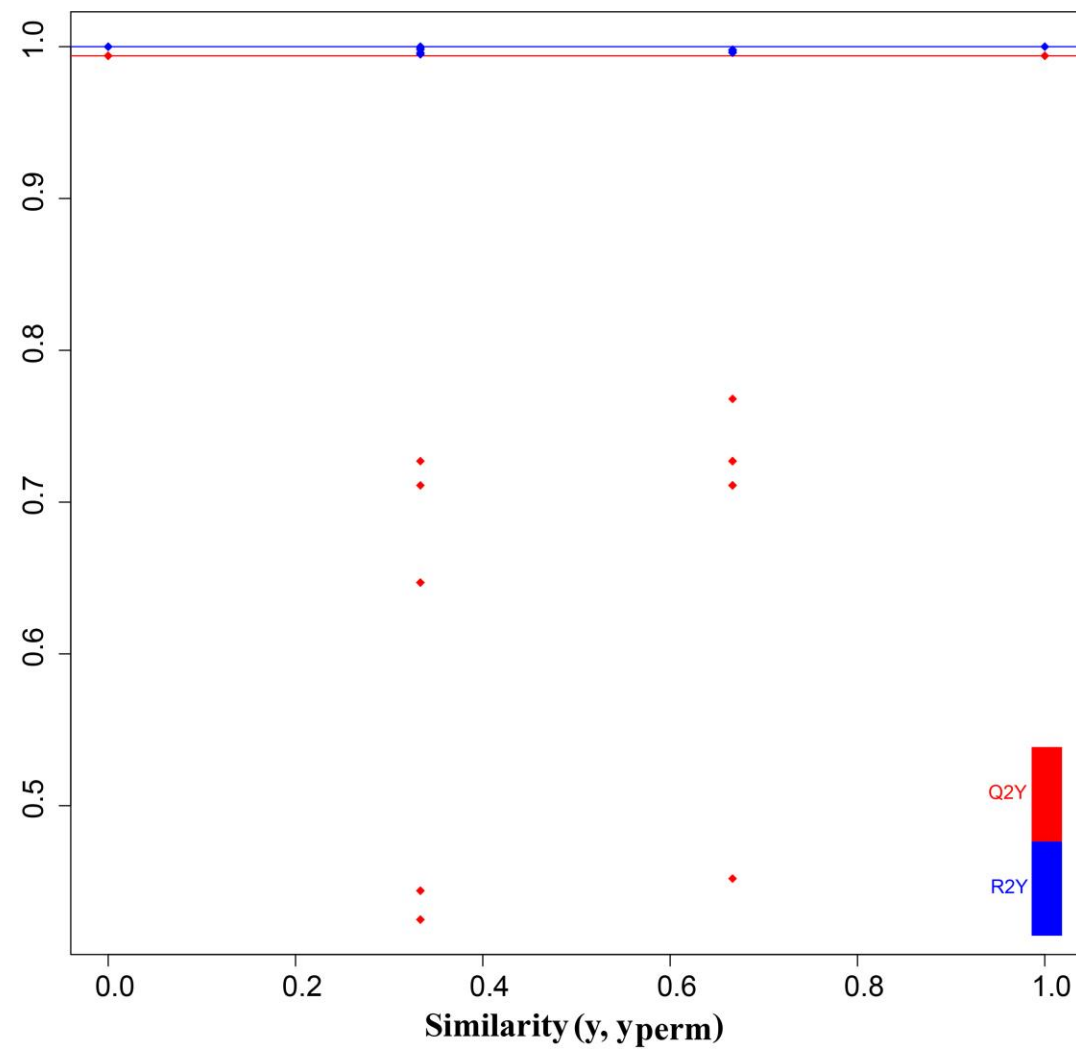

Supplement: Supplementary file 1 [file ijms-22-08751-s001.zip › 5 IJMS-Supplementary Files-8.3/Supplementary Figure S2 Differential metabolites analysis on the basis of OPLS-DA model in leaves of EsMYB90 transgenic tobacco relative to wild type.pdf]
